# Chronic Stereotype Threat Is Associated With Mathematical Achievement on Representative Sample of Secondary Schoolgirls: The Role of Gender Identification, Working Memory, and Intellectual Helplessness

**DOI:** 10.3389/fpsyg.2018.00428

**Published:** 2018-04-03

**Authors:** Sylwia Bedyńska, Izabela Krejtz, Grzegorz Sedek

**Affiliations:** ^1^Department of Psychology, SWPS University of Social Sciences and Humanities, Warsaw, Poland; ^2^Interdisciplinary Center for Applied Cognitive Studies, Department of Psychology, SWPS University of Social Sciences and Humanities, Warsaw, Poland

**Keywords:** stereotype threat, mathematical achievement, working memory, intellectual helplessness, group identitiy

## Abstract

Stereotype threat affects performance in many different groups across many different domains. Despite a large body of experimental research on situational stereotype threat, little attention has been paid to the consequences of repeated experience of stereotype threat. Using structural equation modeling on data from a representative sample of girls from secondary schools, the current research examined the relations of chronic stereotype threat with mathematical achievement, and effectiveness of working memory functions. Moving beyond past theory, this study examined a new mechanism by which chronic stereotype threat decreases school achievement – namely intellectual helplessness. We assumed that repeated experience of stereotype threat works as intellectual helplessness training. After the phase of cognitive mobilization, cognitive exhaustion appears, because the individual has no gain from intense cognitive effort. Corroborating previous research on acute stereotype threat, we demonstrated that chronic stereotype threat is negatively associated with mathematical achievement. Additionally, it was also associated with lower effectiveness of working memory functions, which seems to show depletion of working memory as an effect of chronic stereotype threat. The results also demonstrated that both mediational paths from chronic stereotype threat to mathematical achievement: through working memory depletion and through intellectual helplessness were significant but only for girls that were highly identified with their gender group. In sum, we extended a well-established model of acute stereotype threat to its chronic version and suggested a new mechanism of chronic stereotype threat, which involves intellectual helplessness. Implications for stereotype threat theory and educational practice are discussed.

## Introduction

### Stereotype Threat

The theory of stereotype threat suggests that the activation of negative stereotypes in performance situations reduces the quality of task performance exhibited by group members (Steele and Aronson, 1995). In their seminal work, Steele and Aronson showed that Afro-Americans who were instructed that a test was diagnostic of their verbal abilities solved fewer verbal items of Graduate Record Examination (GRE) than Afro-American students in the control condition in which the test was described as non-diagnostic for verbal abilities. Further research presented diverse and rich evidence from experimental studies on different groups showing that stereotype threat has a significant impact on performance in different domains, e.g., women on math tests (Schmader, 2002), white men when their math abilities are compared to Asian men (Aronson et al., 1999), the elderly on memory tasks (Levy, 1996) (for review see: Schmader et al., 2008). In sum, stereotype threat can negatively affect performance in diverse domains and it is perceived to be responsible for a substantial part of White vs. Latino gap, White vs. Black gap, and gender gap (Walton et al., 2013).

#### Gender Identity as a Moderator of Stereotype Threat

More recently, stereotype threat research has focused largely on moderators and psychological mechanisms underlying the impact of stereotype activation on performance (see review: Schmader et al., 2008). As Schmader et al. (2008) presented, one of the psychological variables defining the susceptibility to stereotype threat is identification with the group that is socially described in terms of stereotypes in a domain (Schmader, 2002). The assumption that those highly identified with their group would be the most sensitive to stereotype threat was supported by the results of an experiment on a sample of female students who solved mathematical section of GRE (Schmader, 2002). Women answered correctly fewer items under stereotype threat than in no-threat condition, but only if they considered gender to be an important part of their social identity. As Schmader explained the results, according to individual’s social identity theory (Tajfel and Turner, 1986) women who are strongly identified with their gender group are also highly motivated to maintain a positive image of that group, and therefore they may experience a higher level of threat and stronger cognitive impairments. In contrast to group identification, Tempel and Neumann (2015) investigated the interplay of gender role orientation and stereotype threat activation. They expected that performance of female students with feminine gender role orientation would be more strongly influenced by stereotype activation in both directions – repressed in a negatively stereotyped domain and boosted in a positively stereotyped domain. The results supported these expectations – femininity was a significant moderator of stereotype influence on both, math performance and emotional sensitivity performance. Under stereotype threat, participants with feminine gender role orientation had lower accuracy in math test, and higher accuracy in emotional sensitivity test. Apart from showing that gender identity and feminine role orientation moderate stereotype threat, the research has also demonstrated that switching group identity helps to avoid negative consequences of stereotype threat (Rydell et al., 2009). By manipulating the availability of different social identities, Rydell et al. showed that changing the identification from gender group to college student group reduced performance deficits in mathematical tasks among females. Thus, they provided support for the hypothesis that group identity is a significant moderator of the effect of stereotype threat on performance.

#### Working Memory as a Mediator of Stereotype Threat

In the past decades, much research has indicated that working memory deficits may be a consequence of stereotype threat manipulation. For example, Schmader and Johns (2003) documented that stereotype threat reduced working memory capacity, as measured by an operational span task which required simultaneous storage and processing of information units. Similar results were obtained using other measures of working memory and its executive functions, such as antisaccade tasks (Jamieson and Harkins, 2007), the Stroop-color naming task (Richeson and Shelton, 2003; Hutchison et al., 2013), or a GO/NOGO task (Mrazek et al., 2011). Furthermore, studies showed that stereotype threat taxes working memory capacity required for successful performance on difficult tasks (Schmader and Johns, 2003; Beilock et al., 2007) and also plays a mediating role between stereotype threat and performance.

Although there is growing evidence that working memory capacity is a significant mediator of the relation between stereotype threat and performance, there are two different accounts that try to explain how negative stereotype activation impairs performance. Schmader et al.’s (2008) integrated process model posits that controlled attention is limited due to the absorption in the active regulation of negative thoughts and negative emotions. Therefore, the working memory capacity is not sufficient for the effective processing of a difficult task. Alternatively, Jamieson and Harkins (2007) mere effort account predicts that participants are hyper-focused on the task because of being highly motivated to disconfirm stereotypes. It is assumed that performance decreases because high motivation prompts greater effort and potentiates one’s dominant response, which might not be efficient to solve new or difficult tasks. As suggested in a recent review (Pennington et al., 2016), both mechanisms, emotional proposed by Schmader et al. (2008) and motivational proposed by Jamieson and Harkins (2007), may operate in distinct ways, depending on the population under study or stereotype threat manipulation. Additionally, attempts to describe the dynamics of reactions to stereotype threat in a similar way to phases of stress coping (Block et al., 2011), suggest that specific mediational processes may be more dominant at different stages of coping with stereotype threat.

#### Acute vs. Chronic Stereotype Threat

Most of the research to date has largely focused on the immediate and temporary impact of stereotype activation on performance and academic achievement as well as the effects of acute stereotype threat (see review: Schmader et al., 2008). However, the consequences of chronic stereotype threat have been progressively gaining on importance (Woodcock et al., 2012; Kalokerinos et al., 2014). There are several studies in the occupational context showing important consequences of chronic stereotype threat. For example, von Hippel et al. (2011) indicated that female employees in male-oriented domains had more negative job attitudes and lower career aspirations to the extent that they experienced stereotype threat. In the same vein, it was demonstrated that older workers experiencing stereotype threat had lower job satisfaction, lower organizational commitment, and in turn higher intentions to resign from work and retire (von Hippel et al., 2013). Furthermore, there was an important link found between chronic stereotype threat experienced in the workplace and negative emotional states such as depression and tension (von Hippel et al., 2013). More recent research has also shown that chronic stereotype threat in female workers is related to higher levels of negative emotions at work, lower job satisfaction, and burnout symptoms such as emotional exhaustion, cynicism, and reduced personal accomplishment (Bedyńska and Żołnierczyk-Zreda, 2015). Much less evidence is provided for educational settings. The only research found in that domain provided strong evidence showing that Latino students who experienced stereotype threat during their scientific career had a weaker intention to persist in science and a lower level of scientific identification (Woodcock et al., 2012).

Comparing emotional consequences of acute and chronic stereotype threat, some similarities can be found. Both types of stereotype threat lead to negative emotions: acute to anxiety (Bosson et al., 2004) and stress-based arousal (Blascovich et al., 2001), while chronic to depression and tension (von Hippel et al., 2013). Acute stereotype threat impairs domain identification (Osborne and Walker, 2006), while chronic is related to domain disengagement and intention to quit (Woodcock et al., 2012). Question arises if, similarly to acute stereotype threat, chronic stereotype threat impairs working memory capacity and achievement. No research has examined the impact of chronic stereotype threat on academic or school performance. Moreover, one may ask if integrated process model of stereotype threat proposed by Schmader et al. (2008), with working memory capacity as a central part of the mechanism, can be also applied to chronic stereotype threat. To our knowledge, the present study is the first one addressing these questions and exploring link between chronic stereotype threat to achievement and working memory as a significant mediator of this link.

Our aim in the present study was not only to present cognitive consequences of chronic stereotype threat but also to propose a new mechanism, based on intellectual helplessness, unique for chronic stereotype threat. Drawing upon past research (e.g., Ståhl et al., 2012) presenting that participants are motivated to engage additional cognitive resources in an attempt to avoid failure under stereotype threat, we posit that this immediate reaction is not beneficial in the long term, as resources are limited. Such cognitive effort without a gain may lead to cognitive exhaustion, as research examining cognitive exhaustion model of learned helplessness demonstrated (Sedek and Kofta, 1990; Kofta and Sedek, 1998). Therefore, we propose a new mechanism that integrates both motivational and cognitive processes involved in a stereotype threat situation. We assume that depletion after mobilization suggested by Ståhl et al. (2012) shows many similarities to the mechanism of intellectual helplessness – a state that is postulated to be a consequence of learned helplessness training at school. In our opinion, as such, intellectual helplessness may be an important mediator of the relationship between chronic stereotype threat and achievement. We now turn to the rationale for this hypothesis by describing intellectual helplessness research.

### Intellectual Helplessness

The roots and manifestations of the phenomenon of intellectual helplessness in relation to school achievement are based on the cognitive exhaustion model of learned helplessness (e.g., Sedek and Kofta, 1990; Kofta and Sedek, 1998). The cognitive exhaustion model has been developed as the cognitive model of experienced uncontrollability and subclinical depression. This approach (e.g., Sedek and Kofta, 1990; Sedek et al., 1993; Kofta and Sedek, 1998; von Hecker and Sedek, 1999) assumes that in typical situations when dealing with problem-solving situations people are likely to engage in a systematic mental activity. They attempt to understand the requirements of a task, notice and pay attention to diagnostic pieces of information, detect regularities or inconsistencies, and so forth. In controllable situations, these mental activities stimulate people to engage in more generative modes of thinking such as the construction of integrative memory representations (e.g., mental models) or the elaboration of complex cognitive strategies with a hierarchy of sub-goals. However, in uncontrollable surroundings (e.g., when the task is not solvable), such an activity is futile because it cannot lead to a real progress in problem-solving. In such situations, a prolonged cognitive effort without cognitive gain results in an altered psychological state, termed “cognitive exhaustion,” characterized by a generalized impairment of more complex processing (Sedek and Kofta, 1990; Kofta and Sedek, 1998; von Hecker and Sedek, 1999). Therefore, after uncontrollable pre-exposure, the ability to generate new hypotheses or solve complex tasks is diminished (Sedek et al., 2010; von Hecker et al., 2013).

Research on intellectual helplessness (Sedek and McIntosh, 1998; Rydzewska et al., 2017) applied the cognitive exhaustion model of learned helplessness to the acquisition of school knowledge. It is assumed that repeated inabilities to understand new material in school despite prolonged mental effort constitute critical conditions for the development of intellectual helplessness. As in laboratory experiments (Sedek and Kofta, 1990; Sedek et al., 1993; Kofta and Sedek, 1998; von Hecker and Sedek, 1999), the prolonged effort with no progress leads to the deterioration in performance when solving cognitively demanding tasks, so such repeated situations during learning in class can block active problem thinking (e.g., involving comparison, reasoning or building consistent knowledge schemas). As demonstrated by previous research, intellectual helplessness is not only related to cognitive deficits (e.g., lower performance in new or difficult tasks), but it is also associated with motivational (e.g., lack of intrinsic and instrumental motivation, disengagement from the domain), and emotional deficits (e.g., anxiety) (Sedek and McIntosh, 1998; Rydzewska et al., 2017).

Integrating the learned helplessness perspective into the body of work on stereotype threat, we propose that repeated experience of stereotype threat (chronic stereotype threat) can produce intellectual helplessness symptoms on a given school domain (e.g., on math). As research showed, participants may be highly motivated to disconfirm a stereotype about theirs group and they mobilize all cognitive resources to solve cognitive tasks in a stereotype threat situation (Ståhl et al., 2012). Because of the working memory deficits, produced by stereotype activation, this cognitive effort does not lead to cognitive gain. Such repeated experience leads to switching into cognitive exhaustion state. As an effect, we can observe performance deficits after pre-exposure to stereotype threat (e.g., lower school grades at mathematics), cognitive deficits such as difficulties in generating mental models (von Hecker et al., 2005) or generating strategy of solving task at hand (Spencer et al., 1999). This mechanism may also link the exposure to stereotype threat with motivational changes: domain disidentification (Osborne and Walker, 2006; Woodcock et al., 2012), lower aspirations (Davies et al., 2005), emotional changes such as a higher level of depression, and a higher level of learned helplessness, a by-product of cognitive exhaustion state. Hence, we assume that the influence of chronic stereotype threat on mathematical achievement is not direct but mediated by intellectual helplessness state and deficits in working memory. The importance and theoretical novelty of this mechanism lies in the possibility of explaining the effects observed in experimental studies on acute stereotype threat such as domain disidentification that are not convincingly explained by Schmader et al. (2008). More importantly, it helps to understand two seemingly contradictory explanations of working memory deficits under stereotype threat, emotional (Schmader et al., 2008) and motivational (Jamieson and Harkins, 2007), as two phases of chronic stereotype threat: mobilization and cognitive exhaustion. In vein with these two lines of studies, we predict that working memory deficits can be observed in both stages of stereotype threat: in the stage of mobilization, when mechanisms of acute stereotype threat are more applicable, and in the stage of depletion (or cognitive exhaustion, as we call this stage), when mechanisms of chronic stereotype threat are more dominating.

To test the above theoretical model we decided to measure chronic stereotype threat in the sample of secondary school females. This was dictated by the fact that girls of this age should have a relatively high level of gender identification and therefore they are more vulnerable to chronic stereotype threat. Our assumption was based on studies showing that gender identification moderated stereotype threat effects on women’s performance in mathematics (Schmader, 2002). Similarly to Schmader (2002), we reasoned that individuals who perceive their own group as important may be more strongly motivated to maintain a positive image of their groups and in consequence may experience stereotype threat more intensely. Additionally, as studies on gender identity development showed (e.g., Kornienko et al., 2016), there is a strong pressure in adolescence from peers and parents to conform gender roles, therefore gender stereotype are probably stronger in secondary school-girls that in later developmental phases. This combination: a strong stereotype endorsement and a strong gender identification may particularly lead to intensive stereotype threat experiences.

### Aims of the Present Study

The central aim of this study was to test the consequences of chronic stereotype threat in a representative sample of secondary school female pupils. We examined the effect of chronic stereotype threat on school achievement measured by school grades in mathematics. We also tested the mediational role of working memory. On the basis of previous research conducted by Oberauer et al. (2000, 2003), three working memory functions were measured: simultaneous storage and processing, coordination, and supervision. Our recent research (Sedek et al., 2016) yielded the evidence that these three functional aspects of working memory are strong predictors of early school achievements. Previous research demonstrated that working memory capacity mediated the relationship between stereotype threat and the performance in a difficult task (e.g., Schmader et al., 2008). We predicted the existence of a similar reliable mediation between chronic stereotype threat and school achievements, when applying as the mediator three aforementioned functional aspects of working memory. We also predicted that there is an additional mediator which may explain the relationship between chronic stereotype threat and academic performance, namely intellectual helplessness. The assumption that intellectual helplessness and the lowered effectiveness of working memory functions independently and significantly predict school underachievement was positively examined in our recent research (Rydzewska et al., 2017). Therefore, we predict that chronic stereotype threat is associated with a higher level of intellectual helplessness and, in consequence, it decreases the effectiveness of working memory and lowers school grades at mathematics. As such, working memory functions and intellectual helplessness were examined as potential mediators in this study. Additionally, gender identification was included as a moderator of chronic stereotype threat effects (Schmader, 2002; Rydell et al., 2009).

## Materials and Methods

### Participants and Procedure

Six hundred twenty four females from gender mixed secondary schools (*M* = 15.59, *SD* = 0.92) took part in the study. The sampling procedure involved two steps. In the first step, 24 secondary schools were randomly sampled with stratification based on region (two regions of Poland) and school location (village, small city, medium city). In the second step, classes were randomly selected from each school and all students belonging to the class were invited as participants (655 female students were selected). Only around 5% of the selected students did not take part in the study due to their absence at school. Study was presented as aimed at testing new online educational games and none of the students resigned from the participation during the study.

The research protocol was approved by the Ethical Committee of the Educational Research Institute. The present study was conducted in compliance with ethical standards adopted by the American Psychological Association (APA, 2010). Accordingly, prior to participation, pupils were informed about the general aim of the research and the anonymity of their data. Participation was voluntary, and the pupils did not receive compensation for their participation in the study. Additionally, parents signed a written consent for their children to participate in the study.

Data was collected in a single session that lasted 45 min, during regular school hours. After explaining procedure and the aim of the study, students solved Functional Aspects of Working Memory Test, and then online questionnaires, with scales of chronic stereotype threat, learned helplessness, gender identity and mathematical achievement among other self-descriptive measures regarding their learning motivation, attitudes toward school and learning. These additional questions were administered before the stereotype threat scale to avoid any potential influence of stereotype threat activation.

### Measures

#### Working Memory

Working memory was measured with Functional Aspects of Working Memory Test (FAWMT), which has been used in educational research and proved to have a high predictive value of early school mathematical achievement (Sedek et al., 2016). The FAWMT was adapted from the battery of experimental procedures used by Oberauer et al. (2000, 2003) and was selected to render the functional complexity of the construct of working memory (Oberauer et al., 2003; Kane et al., 2004).

##### Simultaneous storage and processing function of working memory

Simultaneous storage and processing functions were operationalized with an adaptation of a counting span task, which is the most frequently used measure of children’s working memory capacity (detailed review: Conway et al., 2005). In the counting span task, on each trial the participant was asked to count squares of a target color and ignore both circles of the same color as the target stimuli and squares in a different color than target stimuli. Participants were then asked to recall the number of the target stimuli at the end of each block of trials. The number of trials in a block grew from two to seven. Therefore, the proper completion of the task requires the ability to remember the number of specific elements while processing competing operations (counting target stimuli and inhibiting unnecessary information about other stimuli). A proportion of properly recalled elements in their appropriate order is the basic measure of completing this task.

##### Supervision function of working memory

To operationalize the supervision function, we adopted the set switching task 2 × 2 (Rogers and Monsell, 1995). In the set switching task, the participant is presented with a 2 × 2 matrix in which objects are presented sequentially in a clockwise direction. Each object belongs to two categories and depending on the question asked before the trial participants need to switch alternately between two types of categorization. For example, the participant was asked to classify numbers as odd or even and smaller or bigger than a particular value. There were eight blocks with four trials in each set switching task 2 × 2. The basic measure in this task is the mean proportion of correct classifications.

##### Relational integration function of working memory

Tasks that measure the relational function of working memory, namely relational integration, capture the ability not only to remember a limited number of elements but also to discover the relation between them. As proposed by Oberauer et al. (2003), a spatial location memory task was designed to operationalize this function of working memory. The task required participants to remember the position of an element, colored polygons, as they appeared one by one sequentially on a 10 × 10 matrix. The minimum number of elements in one trial was two, while the maximum was five, which made the last trials very demanding. After each trial, participants were asked to indicate the positions of all presented elements by clicking the computer mouse in the boxes of the matrix. Because of the sequential presentation, in order to solve the task properly the participant had to remember not only the position of all elements but also integrate their location. The overall measure is the mean accuracy of the distance between presented and retrieved elements. A higher accuracy index value can be interpreted as a better memory of the elements’ structure.

#### Intellectual Helplessness

Intellectual helplessness was assessed using selected items from the Intellectual Helplessness Scale (IHS, Sedek and McIntosh, 1998). Students rated to what extent they experience intellectual helplessness symptoms during math class on a 6-point Likert type scale (ranging from 1 – never to 6 – always). The short scale consisted of four statements about feelings and thoughts that accompany math classes: ‘I find I don’t understand what I’m writing in my notes’; ‘It almost takes a physical effort to keep my mind on the lesson’; ‘I feel tired’; ‘I feel helpless’. In the original research (Sedek and McIntosh, 1998), all versions of the scale for various school subjects (math, physics, language) had Cronbach’s Alpha coefficients between 0.92 and 0.95. The short version of the scale was reliable (Cronbach’s α = 0.80).

#### Chronic Stereotype Threat

Chronic stereotype threat was assessed using seven items adapted from Steele and Aronson (1995, Experiment 4) and the Stereotype Vulnerability Scale used in the research in math tests among women (Spencer, 1994). Recently, the same scale was used in longitudinal correlational research on domain disidentification as a long-term consequence of chronic stereotype threat in Latino and African American student samples (Woodcock et al., 2012). We proposed two items measuring specific stereotype threat of being judged by other students (e.g., ‘Other students in my class feel that I have a lower mathematical ability because of my gender’), two items measuring risk of being stereotypically judged by teachers (e.g., ‘I worry that if I fail my teacher will attribute my poor performance to my gender’), and three items measuring generalized stereotype threat (e.g., ‘I worry that if I fail during mathematical test, it will prove that all girls are poor at maths’). Participants answered using a 6-point Likert type scale (ranging from 1 – strongly disagree to 6 – strongly agree). The reliability of the scale was high (Cronbach’s α = 0.89). Construct validity tested using confirmatory factor analysis indicated that the scale was unidimensional (*X*^2^ = 73.556, *df* = 14, *p* < 0.001, *CFI* = 0.928, *TLI* = 0.891, *SRMR* = 0.047, *RMSEA* = 0.083, 90% *CI* (0.065, 0.102), *p* < 0.01).

#### Gender Identity

Gender identity was measured with one item: ‘Being a girl is important to me’. Students rated on a 6-point Likert type scale (ranging from 1 – strongly disagree to 6 – strongly agree) to what extent they agree with this statement. We decided to use only one item to measure gender identity for both theoretical and practical reasons. According to a study on social group identity (Reysen et al., 2013) a single-item measure is a reliable and valid measure of in-group identification. From a practical standpoint, we also wanted to shorten all measurement instruments to avoid tiredness of participants.

#### Mathematical Achievement

Actual Grade Point Average (GPA) in mathematics from two semesters before the study was used as a measure of mathematical achievement. A higher GPA reflects better mathematical achievement with 1 being the failing grade and 6 being the highest grade.

## Data Preparation and Analytical Approach

All analyses were conducted using Mplus 7.3 (Muthén and Muthén, 1998–2015). We used structural equation modeling with complex sampling and the Maximum Likelihood Robust (MLR) approach implemented in Mplus to deal with clustered data (students nested in classes) and a model that contained continuous non-normally distributed variables. This approach takes into account non-independence of observations due to cluster sampling when computing standard errors of the model parameters and a chi–square model fit (Muthén and Satorra, 1995).

In the first step, all classes smaller than three students were excluded from the analysis (seven classes, 15 participants) and 14 students were excluded due to missing values in at least one of the measured variables. In the second step, moderated multiple mediation (Preacher and Hayes, 2008) with one latent variable (working memory capacity) in the structural equation modeling approach (Kline, 2011) was conducted to examine intellectual helplessness and FAWMT as potential mediators of the association between chronic stereotype threat and mathematical achievement. Gender identity was entered as a moderator. Finally, the proposed mediational paths of chronic stereotype threat and mathematical achievement through (1) intellectual helplessness (2) working memory were investigated. We used a 95% confidence intervals (*CI*) method to determine the significance of these indirect effects. This approach is considered as less conservative and lacking in power as compared to the Sobel test (MacKinnon et al., 2002). According to this approach, the indirect effect is significant if the *CI* does not include zero.

All structural models were evaluated using fit indices following Kline’s (2011) recommendations. We used Root Mean Square Error Approximation *(RMSEA)*, Standardized Root Mean Square Residual *(SRMR)*, the Comparative Fit Index *(CFI)*, and the Tucker-Lewis Index *(TLI)* as well as the general fit based on *X*^2^ test of model fit and its associated probability (*p*). We used the most widely recommended cut-off values indicative of an adequate model fit to the data, respectively: *RMSEA* and *SRMR* < 0.06 and < 0.08, *CFI* and *TLI* < 0.95 and < 0.90 (Lance et al., 2006).

## Results

### Descriptive Analysis

The relation between variables included in the model and the associated descriptive statistics are shown in **Table 1**. In line with predictions, mathematical achievement was negatively correlated (*p* < 0.01) with stereotype threat and intellectual helplessness, and positively correlated (*p* < 0.01) with all three functional aspects of working memory. Stereotype threat was only mildly (*r* = 0.20) correlated with intellectual helplessness.

**Table 1:** Means, standard deviations and correlation matrix for the target variables.

	1	2	3	4	5	6	7
(1) Stereotype Threat	–						
(2) Gender Identity	–0.22^∗∗^	–					
(3) Intellectual Helplessness	0.20^∗∗^	–0.03	–				
(4) Storage and Processing	–0.24^∗∗^	0.14^∗∗^	–0.17^∗∗^	–			
(5) Supervision	–0.30^∗∗^	0.10^∗∗^	–0.14^∗∗^	0.44^∗∗^	–		
(6) Relational Integration	–0.21^∗∗^	0.07	–0.13^∗∗^	0.36^∗∗^	0.34^∗∗^	–	
(7) Mathematical Achievement	–0.32^∗∗^	0.06	–0.32^∗∗^	0.29^∗∗^	0.45^∗∗^	0.29^∗∗^	–
Mean	1.986	4.474	4.733	0.693	0.723	0.965	3.147
Standard Deviation	1.084	1.609	0.492	0.191	0.156	0.026	1.009
Range (potential)	1–6	1–6	1–6	0–1	0–1	0–1	1–6

*^∗^p < 0.05, ^∗∗^p < 0.01. ^*a*^Non-standardized path coefficients, ^*b*^Standardized path coefficients.*

### Multiple Mediators Model With FAWMT and Intellectual Helplessness as Mediators

#### Evaluation of the Model

**FIGURE 1:**
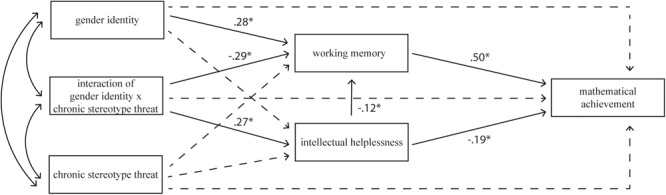
The full mediational model predicting mathematical achievement by chronic stereotype threat with two mediators: functional aspects of working memory, and intellectual helplessness with gender identity as a moderator. (Non-significant paths are indicated by dashed lines, while significant paths are indicated by solid lines; all coefficients are standardized solutions; *p* < 0.05).

Results indicated that the model with intellectual helplessness and FAWMT as mediators (see **Figure 1**) achieved quite a good fit to the data. Although the general test of fit was significant, showing a mediocre general fit *X*^2^ = 20.080, *df* = 10, *p* < 0.05, the inspection of the fit indices values presented a good fit *CFI* = 0.978, *TLI* = 0.946, *SRMR* = 0.018, *RMSEA* = 0.041, 90% *CI* (0.013, 0.066), *p* > 0.05 (*p* = 0.693). The model explained 39.8% of mathematical achievement variability (*R*^2^ = 0.398), 19.1% of the abstract working memory effectiveness (*R*^2^ = 0.191), and 5.5% of helplessness (*R*^2^ = 0.055). The path coefficients are displayed in **Table 2**. All standardized factor loadings for FAWMT were moderate to strong (ranging from 0.495 for Spatial Location Memory Task, 0.598 for Counting Span Task, to 0.733 for Set Switching Task). Data indicates that the majority of the relationships between the variables is consistent with the hypotheses. First, helplessness (β = -0.192, *p* < 0.001) was negatively associated and FAWMT positively associated (β = 0.502, *p* < 0.001) with mathematical achievement. These results indicate that a low level of helplessness and a high level of working memory effectiveness was related to a high level of mathematical achievement.

**Table 2:** Path coefficients in the multiple mediation model with working memory and intellectual helplessness as a mediators.

	*b*^a^	*b*^b^	*SE*	CR	*p*
**Dependent variable: mathematical achievement**					
Intellectual Helplessness → Working Memory	–0.029	–0.124	0.057	2.161	0.031
Stereotype Threat → Working Memory	–0.012	–0.113	0.128	0.882	0.378
Gender Identity → Working Memory	0.019	0.276	0.093	2.976	0.003
Stereotype Threat by Gender Identity → Working Memory	–0.006	–0.288	0.140	2.060	0.039
Working Memory → Mathematical Achievement	4.493	0.502	0.053	9.442	0.001
Stereotype Threat → Mathematical Achievement	0.035	0.038	0.095	0.396	0.692
Intellectual Helplessness → Mathematical Achievement	–0.408	–0.192	0.054	3.573	0.001
Gender Identity → Mathematical Achievement	0.037	0.060	0.077	0.077	0.437
Stereotype Threat by Gender Identity → Mathematical Achievement	–0.034	–0.178	0.112	1.597	0.110
Stereotype Threat → Intellectual Helplessness	–0.002	–0.004	0.105	0.040	0.968
Gender Identity → Intellectual Helplessness	–0.046	–0.156	0.087	1.783	0.075
Stereotype Threat by Gender Identity → Intellectual Helplessness	0.024	0.267	0.112	2.381	0.017

Second, there was a significant relation between FAWMT and its predictors: helplessness (β = -0.124, *p* < 0.05), gender identity (β = 0.276, *p* < 0.05), and the interaction of chronic stereotype threat and gender identity (β = -0.288, *p* < 0.05). Additionally, the interaction term (chronic stereotype threat by gender identity) was a significant predictor of helplessness (β = 0.267, *p* < 0.05). These results confirm that the relationship between stereotype threat and working memory is moderated by gender identity as well as the relationship between stereotype threat and helplessness. This also indicates that learned helplessness is a moderate, but significant, predictor of FAWMT. Additionally, the analysis of the significance of path coefficients revealed that the rest of associations (mostly direct paths) were not significant (see **Table 2**).

#### Indirect Effects of Helplessness and FAWMT as Mediators

The indirect effects of the interaction between stereotype threat and gender identity on mathematical achievement by helplessness and FAWMT performance were assessed using moderated multiple serial mediation models with 95% confidence intervals (Preacher and Hayes, 2008). Three models with indirect effects were evaluated: (1) stereotype threat on mathematical performance mediated only by helplessness, (2) stereotype threat on mathematical performance mediated only by FAWMT, (3) stereotype threat on mathematical performance mediated by sequence of both mediators: learned helplessness and working memory. To test the role of gender identity as a moderator, all models were calculated for a low (-3 *SD*), medium (0 *SD*), and high (3 *SD*) level of gender identity. The results indicated that the potential mediators operated in parallel but not sequentially – two mediations were revealed as significant: mediation by helplessness [β = -0.029, *SE* = 0.013, *p* < 0.05, *CI* (-0.054, -0.004)] and mediation by FAWMT [β = -0.136, *SE* = 0.032, *p* < 0.001, *CI* (-0.198, -0.074)]. Sequential mediation through both helplessness and working memory was not significant ([β = -0.009, *SE* = 0.005, *p* = 0.07, *CI* (-0.020, 0.001)]. All mediators worked only for participants with a high level of gender identity. None of the mediations was significant when gender identity was medium or low.

## Discussion

The main aim of the present study was to test the effects of chronic stereotype threat on school achievement through two mechanisms: a well-known mechanism of acute stereotype threat with working memory as the mediator and a newly proposed mechanism involving intellectual helplessness. Firstly, our results revealed that chronic stereotype threat is related to the lower effectiveness of working memory functions and in turn to the lower mathematical achievement but only in schoolgirls highly identified with their gender group. We found a general pattern of chronic stereotype threat corresponding to low mathematical achievement as measured by school grades. Similar results were also obtained in other studies, indicating that stereotype threat negatively impacted academic achievement, measured by test performance (Schmader et al., 2008) or school grades (Rice et al., 2013).

Additionally, we showed that the negative relation between school achievement and stereotype threat is mediated by three functional aspects of working memory: simultaneous storage and processing, supervision, and relational integration. By including working memory capacity as a mediator and group identity as moderator of chronic stereotype threat we have successfully verified the model of acute stereotype threat proposed by Schmader et al. (2008). We demonstrated that repeated exposure to stereotype threat, leading to chronic stereotype threat, may also cause a persistent depletion of working memory. Thus, our work extends the line of research on working memory deficits being “spilled over” onto subsequent, unrelated to stereotype, tasks (Beilock et al., 2007). It was assumed in these studies that the depletion of resources in one task domain may be carried over to other tasks that require the same processing resources but that are not implicated by the group stereotype. Indeed, Beilock et al. (2007) demonstrated that when stereotype about women being weak at maths was activated, stereotype threat effects occurred in mathematical and verbal tasks when both required phonological aspects of working memory. These performance deficits appeared despite the fact that verbal task was unrelated to gender stereotypes. Our work also provides evidence that not only the impact of stereotype threat may carry over to other tasks related to working memory but also that the effect may be visible across time, producing chronic impairments. Such knowledge contributes not only to theoretical understanding of stereotype threat but also extends the list of environmental factors which can permanently limit working memory functions (Baddeley, 2003). There is a paucity of research on that issue in working memory domain and this work may provide an important input to the research and discussion about models of working memory that mirror the complexity of human cognitive processing.

Secondly, we observed that for participants with a strong gender identification, a higher level of chronic stereotype threat is associated with a higher level of learned helplessness and in turn with a lower mathematical achievement. In essence, the relation between chronic stereotype threat and achievement was mediated by learned helplessness. These results provide an inspiration to experimental studies and a new insight into the mechanism of stereotype threat, suggesting that the repeated experience of acute stereotype threat in school settings is similar to the intellectual helplessness training (Sedek and Kofta, 1990; Kofta and Sedek, 1998; von Hecker and Sedek, 1999). Although there is a clear need for additional research, this theoretical mechanism may offer a plausible explanation as to why stereotype threat leads to domain disidentification and disengagement as well as performance deficits. It would also open possibilities to propose a new type of intervention aimed at reducing stereotype threat effect.

This novel perspective implementing intellectual helplessness into chronic stereotype threat mechanism does not only provide insights into cognitive and motivational consequences of chronic stereotype threat but also reveals new circumstances in which intellectual helplessness can occur. This issue has not yet received adequate attention in the helplessness literature but is important for researchers interested in integrating intellectual helplessness body of work with clinical studies on depression. The latter points to gender differences in depression symptoms (Piccinelli and Wilkinson, 2000; Nolen-Hoeksema, 2001) but there is a lack of information about gender as being substantial moderator of intellectual helplessness – an experimental model of depression. Implementing gender identification as well as processes of stereotyping as the moderators of intellectual helplessness in STEM domains would provide an interesting integration of clinical and social psychological research, bridging these two domains. Seeking a parallel in mechanisms of stereotype threat and intellectual helplessness might be as inspiring as presenting the similarities between depression and stereotyping (Cox et al., 2012). From a practical perspective, it may also lead to development of new psychological interventions to reduce acute and chronic stereotype threat effects.

Thirdly, the purpose of this work was also to examine chronic stereotype threat effects in secondary schoolgirls sample and test gender identification as a moderator of chronic stereotype threat effects. We found that the link between chronic stereotype threat and cognitive deficits was present only in the case of highly identified girls, a finding which corroborates the previous results on acute stereotype threat (e.g., Schmader, 2002). Interestingly, gender identity was not related to chronic stereotype threat. Therefore, we suggest that an experience of chronic stereotype threat is rather weakly determined by identification with their gender group. However, our results demonstrate that gender identification may enhance intellectual helplessness and by this the association may increase cognitive deficits under chronic stereotype threat. This is an issue that should receive more attention in further studies which would integrate these findings with research on the role of social identity in depression (e.g., Cruwys et al., 2014).

### Study Limitation and Future Research

Four limitations of this study should be noted. First, descriptive statistics (means and standard deviation range) suggest that there is almost a floor effect for the level of stereotype threat in this sample. The mean is rather low, and there is not much variance that can explain dependent variables and be related to mediators. However, this may contribute rather to a higher level of type I error than type II error and thus statistically significant relations, even when weak, should be treated as trustworthy. Second, school achievement was measured as actual Grade Point Average. Although some studies showed a strong correlation of GPA and standardized tests (Skórska et al., 2014; Brown et al., 2015), teachers’ expectations and beliefs may strongly contaminate this measure. Thus, other measures of school achievement, such as standardized final or semester tests, should be employed in further studies. Third, the major limitation of the study lies in the type of research design that was used. We used a cross-sectional design to examine the effects among the variables within a path analysis model. However, to establish a cause-effect relationship, a temporal sequence between two variables is needed, which is only possible to test in longitudinal studies. Fourth, only one moderator, namely gender identification, was included in the study, while stereotype threat literature posits several others, e.g., domain identification (Aronson et al., 1999; Keller, 2007), the level of achievement (Régner et al., 2016), stereotype endorsement (Schmader et al., 2004) or test anxiety (Tempel and Neumann, 2014). As Steele and Aronson (1995) stated, the knowledge about this type of variables is not only important for defining vulnerability to stereotype threat but also for the exploration of differences in the mechanism of this phenomenon in different subgroups such as high identifiers with the domain, or with their own group. Future research should consider addressing these limitations.

### Theoretical and Practical Implications

Despite the above limitations, this research has some important practical implications. The relation between chronic stereotype threat and lower working memory capacity can be interpreted as an evidence for stability in impairments of working memory capacity. As working memory was indicated to be important moderator of stereotype threat (Régner et al., 2010), chronic stereotype threat through working memory changes may create a self-sustaining and growing vicious circle. Individuals with a higher level of stereotype threat may be more vulnerable to situational stereotype activation because of stable deficits in working memory capacity.

From a practical perspective, it is essential for teachers and school administrators to be sensitized to the effects of chronic stereotype threat and its consequences. Our research shows that chronic stereotype threat can be measured and it may be related to intellectual helplessness symptoms, the lower effectiveness of working memory functions, and in consequence lower grades. Drawing from our model, it may be assumed that intellectual helplessness mechanism offers a link between stereotype threat, domain disidentification and the loss of interest in mathematics. Therefore, chronic stereotype threat may be, through intellectual helplessness, partly responsible for “leaky pipeline” – progressive under-representation of females in STEM that is persistent despite remedies (Steffens et al., 2010). As the intellectual helplessness syndrome is related to depression symptoms, it is important to further examine the link between intellectual helplessness and negative emotions, such as sadness, anxiety and grief. However, it can also help to establish intervention programs aimed at reducing the influence of chronic stereotype threat on emotions, cognition, and performance by interventions developed to deal with depression. Mindfulness training or methods used in cognitive-behavioral therapy may be suitable to reduce the emotional and cognitive outcomes of a repeated experience of stereotype threat and be a significant preventive method. Such interventions can be easily provided, not only by clinical psychologists but also by teachers as parts of classroom training.

## Ethics Statement

The research was accepted by the Ethics Committee of Educational Research Institute, Warsaw, Poland. Each participant was informed at the beginning of the study about the goal of this study and the computerized procedure, as well as questionnaires to be completed. There was no deception involved in the procedures of the study. The participants and their parents were informed that the participation in this study is voluntary and that they could terminate participation in the study at any moment without giving any rationale. All participants (and their parents) gave informed consent regarding in all of the study procedures.

## Author Contributions

SB: supervision of the project, design of the research, advanced data analysis and interpretation, revising the work, final approval of the version to be published, agreement to be accountable for all aspects of the work. IK and GS: design of the research, basic data analysis and interpretation, article drafting, final approval of the version to be published, agreement to be accountable for all aspects of the work.

## Conflict of Interest Statement

The authors declare that the research was conducted in the absence of any commercial or financial relationships that could be construed as a potential conflict of interest.
